# Prolonged Inhalation Exposure to Coal Dust on Irradiated Rats and Consequences

**DOI:** 10.1155/2022/8824275

**Published:** 2022-02-02

**Authors:** Laura Chulenbayeva, Oralbek Ilderbayev, Dametken Suleymeneva, Aizhan Kaliyeva, Symbat Kabdykanov, Madiyar Nurgaziyev, Ayaulym Nurgozhina, Shynggys Sergazy, Samat Kozhakhmetov, Almagul Kushugulova

**Affiliations:** ^1^Private Institution “National Laboratory Astana”, Nazarbayev University, Nur-Sultan, Kazakhstan; ^2^L.N. Gumilyov Eurasian National University, Nur-Sultan, Kazakhstan; ^3^Semey Medical University NAO, Semey, Kazakhstan

## Abstract

The purposes of this study were to research immune system changes and liver and lung tissues in irradiated rats after prolonged exposure to coal dust. A study was carried out on 30 male Wistar rats that were divided into 3 groups: group I, intact animals; group II, exposure to coal dust and 0.2 Gy *γ*-irradiation; and group III, combined exposure to 6 Gy *γ*-irradiation and coal dust. The combination of a low and sublethal dose of *γ*-irradiation with coal dust leads to a significant change in immunity at the remote period. Particularly, the increase in radioactivity at the combined effect causes weakening of phagocytosis, and reduction in T lymphocytes by a factor of 2, immunoglobulin imbalance, and cytokine dysfunction develop secondary immune failure. During prolonged inhalation with coal dust of irradiated animals with the dose of 0.2 Gy, fibrosis and perivascular sclerosis of the bronchial wall of the lungs are formed, and perivascular fibrosis is formed in the liver. The increase in exposure dose up to 6 Gy in combination with coal, in the distant period, caused pulmonary hypertension amid hypertrophy of light arterial vessels and fibrous changes in arteriole, and destructive changes and collection necrosis develop in liver parenchyma. In the case of dust radiation synergy, the increase in doses leads to a significant immune deficiency, which occurs according to the “dose effect” principle; increases damage to animal tissues; and leads to liver tissue necrosis, pulmonary fibrosis, and pulmonary hypertension.

## 1. Introduction

In the modern reality of our country, one of the most important and pressing issues is to study the consequences of 40-year (1949–1989) nuclear explosions at the Semipalatinsk Nuclear Test Site [1]. As it is well known, throughout many years, the impact of radiation after the repeated explosions has caused instability of ecological, sanitary, medical, and biological conditions of almost all regions of East Kazakhstan and a significant territory of Karaganda and Pavlodar regions (Kazakhstan) [2–4].

It should be emphasized that one of the most harmful factors negatively affecting human health is industrial dust [5]. On the territory of the republic, along with the intensive growth of coal production, there is a tendency to increase hazardous and harmful production factors, which cause harm to the health of the workers [6]. It is commonly known that coal dust is notably different in its chemical composition, particle size, and radioactivity. The main component of industrial dust is silicon dioxide, which is known to develop silicosis in the body. Silicosis is one of the severe forms of occupational diseases related to acute inflammation of the respiratory tract, most often characterized by pulmonary fibrosis and chronic bronchitis. When dust is inhaled, mucosal metaplasia appears in response to inflammatory signals. This is facilitated by the production of T cells, interleukin-6 (IL-6), interferon-gamma (IFN-*γ*), and other ILs closely related to the mandatory inflammatory process; i.e., the activation of inflammatory cells is stimulated. Here, tumor necrosis factor-alpha (TNF-*α*) inflammation activates the recruitment of lymphocytes, neutrophils, and eosinophils, stimulates the expression of chemotoxic factors, and increases the growth of fibroblasts important for the development of fibrosis. IL-6, together with TNF-*α*, is involved in autoimmune processes and is involved in the fibrotic response [7]. Prolonged inhalation causes metabolic disorders, immune system dysfunctions, cytotoxic anomalies, and changes in lung tissue structure. Consequently, all this leads to respiratory diseases [8–10].

Air pollution of the industrial estate of coal production is a serious pathogenic factor that threatens the worker's health, causing the development of chronic inflammatory processes in the lungs and liver [11]. Long-term exposure to coal dust causes the development of industrial pneumoconiosis in coal industry workers, accompanied by the manifestation of chronic bronchitis, chronic obstructive pulmonary diseases, and emphysema with progressive massive fibrosis of the lung tissue [12–15].

Radiation leads to changes in lymphoid cell composition and function in the immune system, resulting in the loss of T-cell immunity. In particular, radiation may induce exhaustion of T-cell functions as depressions in mitogen-dependent proliferation and interleukin-2 (IL-2) production, decrease in helper T-cell populations, and increase in blood inflammatory cytokine levels [16]. Ionizing radiation has a dose-dependent effect on tissues, and with an increase in the radiation dose, vascular damage increases, which leads to tissue necrosis; accumulation and activation of macrophages promote the development of hypoxia. This, in turn, is closely related to changes in the function of the immune system, in particular the cytokine profile, which supports the nonhealing tissue response [17]. The event also is known that liver tissue has a low tolerance to ionizing radiation. Thus, according to Japanese researchers, the victims of the atomic bombings in Nagasaki and Hiroshima were found to have chronic liver diseases, as well as a high risk of death from complications of cirrhosis of the liver [18].

Overall, in the working environment, all harmful factors act in a complex way, causing a combined negative effect on living organisms [19]. According to data, separate effects of the two harmful factors investigated in the body cause significant compound violations and risks, which have been proved by numerous clinical studies and experiments.

It is obvious that in some countries, including Kazakhstan, the population living near the nuclear test site was exposed to radiation, and many of these residents work in coal mines (Karazhyra, Karaganda Coal Basin). In addition, people living in the uranium mining area also work in coal mine. In accordance with this, the practical experience of the research work is to consider what changes occur in organisms exposed to the double factor at a later stage.

In this regard, this work is devoted to the study of the most significant links in the pathogenesis of the toxic effect of coal dust in combination with radioactive exposure to laboratory animals.

## 2. Materials and Methods

### 2.1. Experimental Animal

To achieve this goal, experiments were conducted on 30 white male laboratory rats of the Wister line, weighing 220 ± 20 g, which were divided into 3 groups: group I—intact animals, group II—animals exposed to single dose of 0.2 Gy of *γ*-radiation, subsequently inhaled coal dust of an average concentration of 50 mg/m^3^, and group III—animals exposed single dose of 6 Gy of *γ*-radiation, subsequently inhaled coal dust of an average concentration of 50 mg/m^3^. The experiment was conducted in accordance with ethical standards, according to a meeting of the local ethics committee (Protocol of the Local Ethics Committee of Semey State Medical University, Kazakhstan, No. 2 dated 18.11.2016). On a regular basis, the general condition of the animals, appetite, intensity and nature of motor activity, coordination of movements, the presence and nature of seizures, and the condition of the hair and skin were recorded.

### 2.2. Gamma Irradiation

The animals were irradiated in a TERAGAM ^60^Co radiotherapy device (ISOTREND spol. s r.o.) with a single dose of 0.2 Gy and 6 Gy before the start of the experiment. Before irradiation, experimental topometric and dosimetric preparation of the animals was carried out: the object was placed on an isocentric therapeutic table of the TERASIX X-ray simulator (Czech Republic), which in its design and parameters corresponds to the therapeutic table of a gamma emitter. The cut of the pattern of the irradiated animals was made manually, and then, the topometric data, after being displayed on the display screens, were directly entered into the planning system through the computer's network connection using a digitizer. The isodose calculation was made using the Plan W2000 planning system. Thus, we obtain a topometric-dosimetric map with technical parameters and planned radiation doses for experimental animals. The experimental rats were exposed to general gamma radiation with low and sublethal doses: (1) radiation dose of 0.2 Gy : SSD—97.2 cm, SAD—100.0 cm, area—40 × 40 cm, and *t* = 12 sec; (2) irradiation at a dose of 6 Gy : SSD—97.2 cm, SAD—100.0 cm, area—40 × 40 cm, and *t* = 352 sec (SSD—distance from the source of ionizing radiation to the conditional center of the pathological focus in the device; SAD—distance from the source of ionizing radiation in the device to the nearest irradiated object). During irradiation, the animals were kept in a specially designed organic glass cage with insulated cells for each animal.

### 2.3. Exposure to Coal Dust

To reproduce experimental anthracosis in animals, a special inhalation chamber was used. The animals inhaled coal dust for 90 days with an average concentration of 50 mg/m^3^ daily for 4 hours [20]. The reasons for determining the dose of 50 mg/m^3^ coal for 4 hours per day are remodeling of silicosis in laboratory animals by industrial dust. The animals were placed into cone-shaped compartments, with their vertices attached to the sidewalls of the exposure chamber. The inhalation exposure device is allowed to uniformly disperse the coal dust in the breathing area and maintain the required dust concentration in the chamber with the help of an automatic analyzer.

The device for inhalation of experimental animals with coal dust allows dust to be sprayed in the inhalation chamber, evenly distribute into the animal's breathing zone, and maintain a given concentration of coal dust in the priming chamber using an automatic analyzer. The coal dust used in the experiment was preliminarily crushed on a vibrating crusher. The final fine-tuning to values close to the dispersion of aerosols in the air of the working areas was done manually in an agate mortar. The experiment used coal from the Shubarkol coal mine (Nurminsky District, Karaganda Region in the Republic of Kazakhstan), which is produced by an open method, is characterized by low ash content, and belongs to brown, hummus, and bituminous coals. By chemical composition, the average content of trace elements is as follows: Sc—042 г/т; Cr—3.2; Co—1.9; Zn—22.8; As—0.63; Rb—6.8; Sr—30; Cs—0.03; Ba—7; Au—4.3; U—0.17; Th—0.12; by oxide composition: SiO_2_—60%; Fe_2_O_3_—9%; Al_2_O_3_—24%; CaO—2%; MgO—1.9%; and Na_2_O_3_—0.9%. According to the data, Shubarkol coal is environmentally friendly, and in terms of trace element composition, it belongs to aluminosilicate (SiO_2_ and Al_2_O_3_) coal and the concentration of radioactive elements in coal is low [21, 22]. Since the content of silicon dioxide in coal dust is from 10 to 70%, our used coal belongs to the 4th hazard class (low hazard).

Special restrictions are imposed on the amount of coal dust in the environment, and according to the US Federal Law on safety and protection in coal mines of 1969 (Coal Law), the maximum permissible concentration (MPC) of dust on the surface and under the ground should not exceed 2 mg/m^3^ and the content of silicon dioxide, one of the toxic components, should not exceed 50 mg/m^3^.

### 2.4. Immune Parameters

#### 2.4.1. Measurement of a Subpopulation of Lymphocytes

In all experimental animals, the total number of leukocytes and lymphocytes in the peripheral blood was determined. The number of B and T lymphocytes and their subpopulations was determined by the method of immunofluorescent staining of cells using antibodies conjugated to fluorescein isothiocyanate (FITC). The following monoclonal antibodies were used: CD3+, CD4+, CD8+, CD20+ FITC (BD Biosciences). The reaction of inhibition of leukocyte migration (LMIT) and the concentration of circulating immune complexes (CIC) was determined.

#### 2.4.2. Measurement of the Cytokine Profile

The content of pro-inflammatory cytokines IL-2, IL-6, TNF-*α*, and IFN-*γ* was determined in the blood serum by quantitative enzyme immunoassay on the equipment “Uniplan” (Russia). The test system for the determination of cytokines in experimental animals (specifically for rats: rat IL-2, rat IL-6, rat TNF-*α*, and rat IFN-*γ*) was delivered from Austria, “Bender MedSystems” (Austria) with the assistance of CJSC “BioChemMac.” To calculate the concentration of the studied cytokines, standard calibration dilutions and software supplied by “Bender MedSystems” (FlowCytoMix) included in the kit were used.

### 2.5. Tissue Histomorphology

Lung and liver tissues from experimental rats were removed during autopsy for histopathological examination. Tissues were subjected to conventional histological processing with the preparation of paraffin sections of 5–7 microns thick, followed by their staining with hematoxylin and eosin.

### 2.6. Statistical Analysis

Analyses were performed using *R* studio version *R* 3.6.2 software. Mean differences were compared using a one-way ANOVA, and subsequent pairwise comparisons were performed using Tukey's test. The Kruskal–Wallis test is used for multiple comparisons of continuous variables with nonnormal distribution. *P* < 0.05 was considered statistically significant.

## 3. Results

We investigated a subpopulation of lymphocytes and cytokine production in the peripheral blood of rats after combined and separate exposure to coal dust and sublethal *γ*-ionizing radiation in the long term. When assessing the functional state of the immune system (Supplementary Table 1) and the histomorphology of the tissues of the lungs and liver, a number of changes in indicators were observed describing the disturbances by the influence of the studies generating environmental factors. The general condition, appetite, behavior, and possible death of the animals were monitored throughout the experiment. During 90 days after a single irradiation of 0.2 Gy and 6 Gy with a dose of irradiation and subsequent inhalation with coal dust during the experiment, no changes were observed in the behavior, motor activity, and appearance of laboratory animals.

### 3.1. Immune System Сhanges

**Figure 1:**
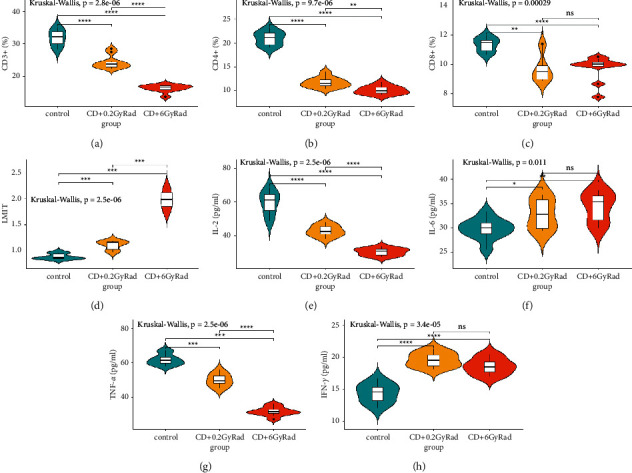
T-cell immune response to combined effect of coal dust and radiation: (a) CD3+; (b) CD4+; (c) CD8+; (d) LMIT; (e) IL-2; (f) IL-6; (g) TNF-*α*; (h) IFN-*γ*.

**Figure 2:**
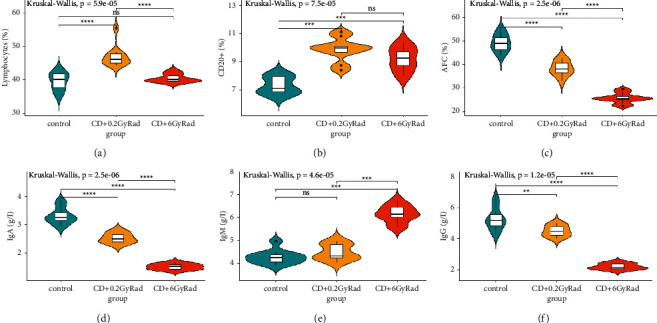
B-cell immune response to combined effect of coal dust and radiation: (a) lymphocytes; (b) CD20+; (c) AFC; (d) IgA; (e) IgM; (f) IgG.

In irradiated animals with 0.2 Gy ionizing radiation, after inhaling coal dust for a long time, cellular CD3+ T lymphopenia was observed due to the activation of CD4+ lymphocytes by 30.4% (*p* < 0.0001) and CD8 lymphocyte subpopulations by 26.3% (*P* < 0.001), and as a consequence, the repression of the leukocyte migration inhibition by 24.71% (*P* < 0.001) occurs, which leads to a deterioration in the functioning of immune cells. In addition, the effect of the combined factor also changed the production of cytokines in the long-term period; that is, the levels of IL-2 (1.4 times, *P* < 0.001) and TNF-*α* (1.2 times) (*p* < 0.0001) decreased and against increased IL-6 (0.001) and IFN-*γ* (1.4 times, *p* < 0.0481) (Figures 1(a)–1(h)). Despite the occurrence of lymphocytosis due to CD20+, a decrease in the level of immunoglobulins IgA (23.6%, *P* < 0.0001) and IgG (*p*=0.0032) and antibody-forming cells (AFCs) in the spleen leads to the incomplete performance of the function of humoral immunity. However, significant stimulation of the nonspecific phagocytic link of immunity has been registered. There are suggestions that stimulation of the phagocytic activity of leukocytes guarantees an increase in the phagocytic protective function of the body. This process contributes to a decrease in the function of the T and B cells of immunity (Figures 2(a)–2(f)).

The combined effect of ionizing radiation at a dose of 6 Gy and long-term coal dust leads to a decrease in the indicators of the CD4-positive T cells by 2.3 times, CD3+ by 3.02 times (*p* < 0.0001), and CD8+ lymphocyte subpopulations by 1.7 times (*p* < 0.001), and in the peripheral blood due to CD4+/CD8+ ratio, the immunoregulatory index is inhibited by 25.9% (*p* < 0.0001); on the contrary, the stimulation of leukocyte migration inhibition test (LMIT) due to general lymphocytosis leads to a weakening of cellular immunity. At the same time, the suppression index (SI) increased by 47.76% (*p* < 0.0001), which shows the development of the II degree inflammatory process. Long-term exposure to sublethal *γ*-radiation and coal dust damages the production of pro-inflammatory cytokines IL-2 (*p* < 0.0001) and TNF-*α*(*p* < 0.0001) by 48.5% and activates the synthesis of cytokines IL-6 (*p* < 0.003) and IFN-*γ* (*P* < 0.001) (Figures 1(a)–1(h)). The combined effect of coal dust and radiation at a dose of 6 Gy in the long-term period subsequently leads to violations of the humoral links and the mononuclear-phagocytic system of immunity, namely, to a change in local immunity with an insignificant synthesis of a representative of B-cell CD20+ lymphocytes by 13.95% (*p*=0.05) and IgA and IgG proteins by 2.2 times (*p* < 0.0001) and 2.4 times (*P* < 0.001), respectively (Figures 2(a)–2(f)). In addition, a profound insufficiency of the phagocytic activity of the organism was registered. It is known that the weakening of the activity of phagocytosis caused by ionizing radiation forms a leukopenic form of phagocytic insufficiency, which, in turn, develops as a result of slowing down the processes of proliferation and/or maturation of leukocytes [23, 24].

When comparing the parameters of the immune system between groups of animals that received the combined effect of coal dust with a low dose of *γ*-irradiation (group II) and a high dose of *γ*-irradiation (group III), significant changes were revealed. Almost all indicators were reduced in animals exposed to the combined effect of coal dust and 6 Gy *γ*-irradiation. On the contrary, LMIT stimulation and an increase in suppression index levels were detected. No significant deviations were observed in the IL-6, IFN-*γ*, and CD20 + values.

### 3.2. Histopathological Examination of Tissue

The results of the histology material morphological examination of the intact rats showed that in the liver and lungs tissues developed the same type of moderately expressed changes.

**Figure 3:**
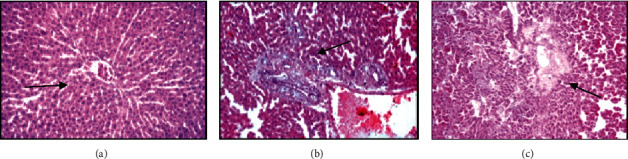
Histological structure of the liver; staining with hematoxylin and eosin. (a) Intact group: the grinder structure of the hepatic lobules is preserved, moderate plethora of the central vein and moderate expansion of sinusoids, zoom x100; (b) rat liver histology after 90 days after dose of 0.2 Gy single irradiation in combination with daily exposure to coal dust for 4 hours: in the interlobular stroma of the proliferation of fibrous fibers around the components of the triad and moderate infiltration with lymphocytes and histiocytes, plethora of the dilated vein, zoom x100; (c) rat liver histology after 90 days after dose of 6 Gy single irradiation in combination with daily exposure to coal dust for 4 hours: foci of colliquation necrosis in the paracentral zone of the hepatic lobule and discomplexation of the hepatic tracts, zoom x200.

*Liver.* Intact rats liver tissue retained a lobular and girder structure with a moderate expansion of sinusoids and a moderate plethora of central veins. In the periportal area signs of moderate edema, accumulations of single lymphocytes were determined; the portal triad (bile duct, arteriole, and venous vessel) showed no pathological changes (Figure 3(a)).

Rat liver of the second series observed microcirculation disorder with plasma dissociation from blood cells against the background of diffuse protein degeneration of hepatocytes, uneven expansion, and a plethora of sinusoids, and the lumens of small vessels were filled with plasma proteins. Periportal area was indicated of fibrosis and moderate lymphocytic-histiocytic infiltration, a pronounced plethora of dilated veins (Figure 3(b)).

In the liver of animals from group 3 were observed diffuse protein dystrophy and swelling of hepatocytes, moderate fibrosis, and lymphocytic and histiocytic infiltration in the periportal area. Alongside these changes, foci of hepatocyte colliquative necrosis and discomplexation of hepatic beams in some hepatic lobules of paracentral area were revealed (Figure 3(c)).

**Figure 4:**
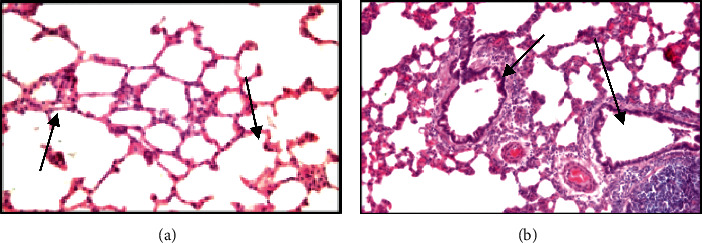
Histological structure of the lungs of intact rats: (a, b) staining with hematoxylin and eosin. (a) Emphysematous enlargement of the alveoli; uneven moderately expressed thickening of the interalveolar septa against the background of vascular congestion and edema, zoom x100. (b) Lymphocytic infiltrates in the interalveolar septa, around the congested vessels and bronchi; bronchi with preserved contours and moderate secretory activity of the epithelium, zoom x160.

*Lungs.* The results of the histology material morphological examination of the intact rats showed that moderately expressed changes in the same type develop in the tissue of the lungs and liver of rats. Thus, in the lungs of intact group, rats revealed feature of irregular emphysema distension in alveoli and uneven thickening of the interalveolar septa due to a moderate plethora of blood vessels and edema, unevenly expressed lymphocytic infiltration of interalveolar septa, and peribronchial and perivascular spaces. The bronchi retained their contours, and the bronchial epithelium showed a pronounced moderate activity of mucus production (Figures 4(a) and 4(b)).

In rats of the second series, 90 days after the beginning of the experiment, histological signs of fibroblastic process activation in presence of interstitial inflammation of the lungs and liver were found.

Thus, in the lung tissue, along with inflammatory lymphocytic infiltration of the bronchial wall, peribronchial tissue, and interalveolar septa, pronounced fibrous changes in the bronchial wall and perivascular and peribronchial tissues were revealed. In addition, in the lungs of experimental animals, the phenomena of bronchial ectasia with cystic enlargement of the lumens and flattening of the bronchial epithelium along the length, against the background of inflammation and fibrosis of the bronchial wall, were noted (Figures 4(a) and 4(b)).

In experimental animals of the third experimental series, 90 days after the beginning of the experiment, against the background of signs of interstitial inflammation and fibroblastic changes in the lung tissue, pronounced changes in the bronchi and vascular walls were observed.

**Figure 5:**
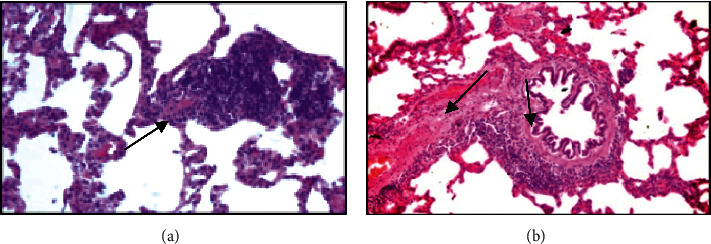
Histological structure of rat lungs 90 days after a single irradiation at a dose of 0.2 Gy in combination with exposure to coal dust every day for 4 hours. (a) Interstitial pneumonia in the form of pronounced lymphocytic infiltration of interalveolar septa, zoom x160; (b) inflammatory lymphocytic infiltration and pronounced fibrosis of the bronchial wall; peribronchial and perivascular tissues of the lung stroma, zoom x160.

In the bronchi, signs of a focal proliferation of epithelial cells of the mucous membrane with hypersecretion of mucus were noted; severe lymphocytic infiltration of the bronchial wall and peribronchial tissue unevenly expressed hyperplasia of the peribronchial lymphoid tissue (Figures 5(a) and 5(b)).

**Figure 6:**
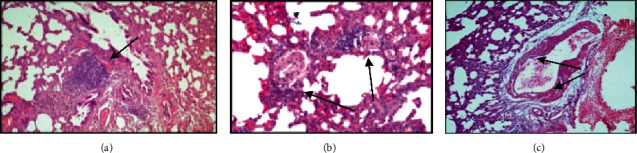
Histological structure of rat lungs 90 days after a single irradiation at a dose of 6 Gy in combination with exposure to coal dust every 4 days for 4 hours: staining with hematoxylin and eosin. (a) Hyperplasia of peribronchial lymphoid tissue against the background of peribronchial and perivascular fibrosis of the lung stroma, zoom x180; (b) fibrosis of the arteriole wall in the lung interstitium, perivascular infiltration with lymphocytes, zoom x160; (c) hypertrophy of muscle-elastic fibers in the wall of the arterial vessel of the lung stroma, zoom x200.

Changes in the vascular wall in the lung interstitium were expressed in fibrinoid changes and fibrosis of the arteriole wall and hypertrophy of muscular-elastic fibers in the wall of arterial vessels. Fibrinoid changes in the vascular wall of the microcirculatory bed were accompanied by the formation of lymphoid cell “sleeves” around arterioles and capillaries, in places having the form of lymphoid cell granulomas (Figures 6(a), 6(b), and 6(c)).

## 4. Discussion

The phenomenon of cellular T lymphopenia CD4+, CD8+, and CD3+ and the associated activation of an inhibitory response to leukocyte migration in animals exposed to coal dust for a long time after ionizing radiation of 0.2 Gy led to a deterioration in the function of immune cells. In addition, the influence of these two factors also changed the production of cytokines in the subsequent period, that is, decreased the levels of IL-2 and TNF-*α* and increased the levels of IL-6 and IFN-*γ*. In addition, despite the occurrence of lymphocytosis due to CD20+, a slight decrease in IgA and IgG immunoglobulins and a weakening of AFC in the spleen lead to incomplete humoral immune function. However, significant stimulation of the nonspecific phagocytic link of immunity was noted. Stimulation of this phagocytic activity of leukocytes guarantees an increase in the phagocytic protective function of the body, although it is assumed that this process contributes to the inhibition of the function of T lymphocytes and B lymphocytes of the immune system.

The combined effect of 6 Gy of ionizing radiation and coal dust suppresses subpopulations of CD4+, CD8+, and CD3+ and reduces the immunoregulatory index (CD4+/CD8+) in the peripheral blood, weakening the T-cell immune link; on the contrary, the activation of LMIT due to general lymphocytosis leads to a weakening of the cellular immunity. The combined long-term exposure to coal dust and sublethal radiation dose disrupts the production of pro-inflammatory cytokines IL-2 and TNF-*α*, causing the excessive synthesis of cytokines IL-6 and IFN-*γ*. The combined effect of coal dust and irradiation at a dose of 6 Gy in the long-term period led to a violation of the humoral status and mononuclear-phagocytic defense system of the immune system; in particular, it increased the number of B-cell CD20+ lymphocytes and reduced the level of immunoglobulins IgA, IgG, and AFC; on the contrary, it activated the synthesis of immunoglobulin IgM, which led to the collapse of local immunity. In addition, there is a deep lack of phagocytic activity of the body.

Long-term inhalation of coal dust in irradiated animals weakens the production of T lymphocytes, suppresses cellular immunity by increasing the generation of B cells, and activates humoral immunity. The effect of ionizing radiation often reduces the function of Тһ1 and leads to disruption of Тһ1/Th2 equilibrium, which shifts towards the priority of Тһ2, and this imbalance during irradiation can affect immune dysfunction [25]. The balance of Th1/Th2 cells is essential for host immunity. In turn, Th1 cells increase the body's resistance to intracellular pathogens and form cytokines such as IFN-*γ*, which is the main cytokine that depends on the host's resistance to viral infections. Th2 cells are resistant to extracellular pathogens and act against the production and activity of Th1 cytokines, including IFN-*γ*. Thus, a change in the Tһ1/Th2 balance can affect various immune-mediated diseases, including allergies, autoimmunity, and increased sensitivity of the body to infections [26].

Single irradiation at a dose of 0.2 Gy in combination with inhalation of coal dust in the tissue of the lungs and liver of experimental rats, against the background of signs of chronic interstitial inflammation, fibroblastic processes develop with the development of chronic bronchitis and bronchial ectasia with fibrosis of the bronchial wall; perivascular sclerosis of the lung stroma; and periportal fibrosis in the interlobular stroma of the liver. Long-term exposure to coal dust after high-dose irradiation in the liver of rats observed that focal destruction of the liver parenchyma is revealed in the form of foci of colliquation necrosis of the subcapsular zones and paracentral zones of the hepatic lobule. Liver fibrosis is caused by damage to the liver by various chronic effects (radiation and coal dust), which disrupt liver homeostasis and increase the production of reactive oxygen species, which are generated by lipid peroxidation [27]. Fibrosis can progress over several months to decades and turn into cirrhosis of the liver, which can lead to the development of liver carcinoma [28, 29].

The data of histopathological studies showed that a single exposure to a small dose of radiation followed by inhalation exposure to coal dust for 90 days causes the development of a morphological picture of chronic bronchitis with bronchiectasis, which is manifestation of chronic obstructive pulmonary disease (COPD) with an outcome in pneumosclerosis [30]. The observed fibroblastic processes are the result of chronic intoxication and immune disorders, and adaptation processes are referred to in the form of incomplete tissue regeneration. Similar changes in the lungs of animals and humans that develop after radiation exposure and lead to radiation pneumonitis and pulmonary fibrosis have been described by a number of authors. For example, in patients receiving radiation therapy, after exposure to a high dose of radiation for two weeks, signs of bronchiectasis, diffuse changes, and pulmonary fibrosis were observed [31, 32]. This is reflected in the production of T cells, IL-6, IFN-*γ*, and other interleukins, which are closely related to the mandatory inflammatory process; that is, the activation of inflammatory cells is stimulated. Here, TNF-*α* activates the recruitment of inflammatory lymphocytes, neutrophils, and eosinophils, stimulates the expression of chemotoxic factors, and increases the growth of fibroblasts, which are important for the development of fibrosis. IL-6, together with TNF-*α*, is involved in autoimmune processes and is involved in the fibrotic response [33]. Also, according to the literature, coal workers with pneumoconiosis have a high incidence of bronchiectasis caused by the duration of inhalation and a high concentration of inhaled coal dust [34].

In the lungs of experimental rats that received a sublethal dose of radiation followed by inhalation of coal dust, the phenomena of the proliferation of the epithelium of the bronchial mucosa with an increase in secretory activity against the background of chronic inflammation were noted. As a manifestation of the dysfunction of the humoral and cellular immune response, signs of hyperplasia of the peribronchial lymphoid tissue and the formation of lymphoid cell granulomas against the background of interstitial pneumonia were revealed in the lung tissue. It is known that the outcome of interstitial inflammation of lung tissue is diffuse pneumosclerosis, often caused by inhalation of inorganic substances from the environment [35].

Hypertrophy of muscular-elastic fibers in the wall of arterial vessels of the lung tissue of rats of group III indicates the development, within 90 days of experimental observations, of pulmonary hypertension against the background of fibrotic changes in the arteriole wall. A circulatory disorder against the background of pneumosclerosis is the cause of the development of pulmonary hypertension. Respiratory hypertension is a rare, chronic, and progressive disease accompanied by narrowing of the lumen of the intrapulmonary vessels, complicating pulmonary and cardiovascular diseases, and, ultimately, leading to death from pulmonary heart decompensation [36]. According to a number of authors, radiation causes pulmonary hypertension regardless of the radiation dose, and morphological changes in the lungs with signs of chronic inflammation, emphysema, and pneumofibrosis develop as early as 2 months after irradiation [37].

## 5. Conclusions

The results of the pathohistological studies carried out indicate that 90 days (long-term period) after receiving the dust and radiation exposure, significant changes in the body of experimental animals were revealed. Both factors individually alter the immune system and cause liver and lung damage. Based on the data obtained, it can be judged that coal dust and radiation enhance the harmful effect of each other.

The combined effect of low and sublethal doses of *γ*-radiation with coal dust in the long-term leads to a pronounced change in immunity. An increase in the dose of radioactivity with a combined effect causes a significant weakening of phagocytosis, a decrease in T lymphocytes by 2 times, an immunosuppressive state against the background of an imbalance of immunoglobulins, and cytokine dysfunction, which contributes to the development of secondary immune deficiency.

Long-term inhalation to coal dust in irradiated animals with a low dose (0.2 Gy) fibrosis and perivascular sclerosis of the bronchial wall of the lungs and perivascular fibrosis in the liver tissues were developed. An increase in the radiation dose to 6 Gy with combined exposure to coal dust in the long-term period caused pulmonary hypertension against the background of hypertrophy of the arterial vessels of the lungs and fibrotic changes in arterioles and in the liver parenchyma the development of destructive changes and colliquation necrosis.

## Data Availability

The data used to support the findings of this study are available on request.

## Supplementary Materials

Supplementary Materials Supplementary Table 1: combined effect of *γ*-irradiation (Rad) and coal dust (CD) on the animal immune system parameters in the long term (90 days) (М ± *m*).
