# Supplementary material for: Prolonged Inhalation Exposure to Coal Dust on Irradiated Rats and Consequences
Source: ScientificWorldJournal. 2022 Feb 2;2022:8824275. doi: 10.1155/2022/8824275 (PMC8828334; doi:10.1155/2022/8824275)
Supplement: Supplementary Materials — Supplementary Table 1: combined effect of γ-irradiation (Rad) and coal dust (CD) on the animal immune system parameters in the long term (90 days) (М ± m). [file 8824275.f1.docx]

Supplementary Table 1: Combined effect of γ-irradiation (Rad) and coal dust (CD) on the animals immune system parameters in the long term (90 days), (М±m)

| Immune parameters | | | Intact (group I) | 0.2 Rad+CD (group II) | 6 Gy Rad+CD (group III) | *P* values | Groups compared |
| --- | --- | --- | --- | --- | --- | --- | --- |
| Leucocytes, ×10^9^ /l | | | 6,66±1,45 | 6,29±0,81 | 4,66±0,5 | *P* < 0.001  *P* = 0.003 | CD+6GyRad-control  CD+6GyRad-CD+0.2GyRad |
| Lymphocytes, ×10^9^ /l | | | 2,86±0,12 | 3,72±0,30 | 2,62±0,25 | P < 0.001 | CD+0.2GyRad-control  CD+6GyRad-control |
| Lymphocytes, % | | | 39,5±2,56 | 47,0±3,22 | 40,4±1,22 | P < 0.0001 | CD+0.2GyRad-control  CD+6GyRad-CD+0.2GyRad |
| CD3+ | abs.number | | 1,54±0,09 | 1,26±0,10 | 0,51±0,05 | P < 0.0001 | Between all groups |
|  | % | | 31,91±2,45 | 24,35±2,06 | 16,31±1,14 | P < 0.0001 | Between all groups |
| CD4+ | abs.number | | 0,79±0,05 | 0,55±0,04 | 0,34±0,03 | P < 0.0001 | Between all groups |
|  | % | | 21,02±1,43 | 11,73±1,07 | 10,15±0,87 | *P* < 0.001  *P* = 0.012 | CD+0.2GyRad-control  CD+6GyRad-control  CD+6GyRad-CD+0.2GyRad |
| CD8+ | abs.number | | 0,57±0,03 | 0,42±0,04 | 0,33±0,03 | *P* < 0.001 | Between all groups |
|  | % | | 11,34±0,46 | 9,71±0,92 | 9,74±0,85 | *P* < 0.0001  *P* < 0.0002 | CD+0.2GyRad-control  CD+6GyRad-control |
| Ratio CD4+/CD8+ | | | 1,38±0,05 | 1,31±0,09 | 0,99±0,11 | *P* < 0.0001 | CD+6GyRad-control  CD+6GyRad-CD+0.2GyRad |
| LMIT | | | 0,89±0,05 | 1,11±0,08 | 1,98±0,17 | *P* < 0.001 | Between all groups |
| CD20+ | | abs.number | 0,43±0,03 | 0,76±0,05 | 0,49±0,03 | *P* < 0.001  *P* = 0.005 | CD+0.2GyRad-control  CD+6GyRad-CD+0.2GyRad  CD+6GyRad-control |
|  |  | % | 7,31±0,60 | 9,87±0,84 | 9,21±0,85 | P < 0.001 | CD+0.2GyRad-control  CD+6GyRad-control |
| AFC, % | | | 49,12±3,57 | 38,33±3,09 | 25,66±2,15 | P < 0.0001 | Between all groups |
| SI % | | | - | 22,6±3,28 | 47,7±4,38 | P < 0.0001 | Between all groups |
| CIC, ед. | | | 1,4±0,06 | 1,26±0,15 | 0,55±0,07 | *P* = 0.0125  *P* < 0.001 | CD+0.2GyRad-control  CD+6GyRad-CD+0.2GyRad  CD+6GyRad-control |
| Phagocytosis, % | | | 36,20±2,57 | 47,5±4,2 | 22,20±1,65 | P < 0.0001 | Between all groups |
| Phagocytic number | | | 1,57±0,21 | 2,02±0,55 | 1,23±0,11 |  |  |
| NTB, % | | | 4,97±0,56 | 7,46±0,64 | 4,77±0,41 | *P* < 0.001 | CD+0.2GyRad-control  CD+6GyRad-CD+0.2GyRad |
| Ig A, g/l | | | 3,31±0,29 | 2,53±0,18 | 1,52±0,10 | P < 0.0001 | Between all groups |
| Ig M, g/l | | | 4,27±0,30 | 4,49±0,35 | 6,15±0,37 | *P* < 0.001 | CD+6GyRad-control  CD+6GyRad-CD+0.2GyRad |
| Ig G, g/l | | | 5,27±0,72 | 4,70±0,34 | 2,21±0,19 | *P* = 0.0032  *P* < 0.001 | CD+0.2GyRad-control  CD+6GyRad-CD+0.2GyRad  CD+6GyRad-control |
| IL-2 (pg/ml) | | | 59,50±6,73 | 42,99±3,21 | 30,66±2,19 | P < 0.0001 | Between all groups |
| IL-6 (pg/ml) | | | 29,77±2,14 | 32,87±3,07 | 34,31±3,02 | *P* = 0.0481  *P* < 0.003 | CD+0.2GyRad-control  CD+6GyRad-control |
| TNF-α (pg/ml) | | | 61,93±3,20 | 50,11±3,67 | 31,86±2,61 | P < 0.0001 | Between all groups |
| IFN-γ (pg/ml) | | | 14,36±1,47 | 19,58±1,12 | 18,56±1,26 | *P* < 0.001 | CD+0.2GyRad-control  CD+6GyRad-control |
| Note: Median values between the groups were compared using the Mann-Whitney *U* test | | | | | |  |  |
